# Where Do All Those Genes Come From?

**DOI:** 10.1371/journal.pbio.0030169

**Published:** 2005-04-05

An important source of genetic novelty is the introduction of new genes. Since most genes in an organism's genome are under selective constraint, opportunities for the evolution of new gene functions—which in turn might confer selective advantage—most often arise when new genes enter the genome. In eukaryotes—a category that includes humans and rice—novel genes typically arise when existing genes undergo duplication. Extra copies of genes can be created when normal DNA replication hiccups and erroneously duplicates entire regions of DNA. These extra gene copies reside in species' genomes for generations and might eventually mutate to code for novel proteins, adding new genes to the species' repertoire. The new genes, along with the rest of the genome, are passed down from one generation to the next in a process known as vertical transmission.

In prokaryotes—which include unicellular organisms in the bacteria and archaea domains—novel genes can appear through multiple routes. In addition to gene duplication, prokaryote genomes can change when DNA fragments are taken up directly by cells, passed from cell to cell, or transferred to new cells with the help of viruses. All three scenarios provide a means for whole genes to move directly from one bacterial genome to another, a process called lateral gene transfer (LGT) or horizontal transmission.

Until now, the importance of vertical versus horizontal transmission in the evolution of any large prokaryote group was unknown. In a new study, Emmanuelle Lerat et al. capitalized on the availability of complete genome sequences within the diverse γ-Proteobacteria, a group of prokaryotes that includes Escherichia coli, Salmonella spp., and some nitrogen-fixing bacteria, to pursue that question.

Sorting out the issue is no simple task. If the same gene is present in more than one species, it could have been inherited from a common ancestor or it could have jumped from one lineage to another by LGT. Even if the same gene appears twice in one species' genome, the copies could have different histories—one copy could have been acquired vertically from its ancestors, while the other could have come from a different species.

Though previous studies have looked at the distributions of genes across species phylogenies, information about gene origin appeared contradictory. To create a clearer picture, Lerat et al. accounted for the possibility of widespread LGT by statistically comparing the phylogenies of many different gene families with a benchmark phylogenetic tree that reflected the accepted evolutionary history of γ-Proteobacteria.

The authors found that LGT plays a substantial role in generating the diversity of genes found in γ-Proteobacteria genomes. Members of the group are constantly acquiring and losing genes, although the extent of LGT can vary greatly among species. In contrast, gene duplications play a much smaller role in explaining γ-Proteobacteria genome diversity, although duplications have been shown to be important for short-term adaptation.

Genes that have arrived by LGT within a single genome do not necessarily share a common history with each other. Many of the genes that are found only in a single genome and are not widely distributed across the γ-Proteobacteria were recently acquired from distant sources. Most of these acquired genes will likely be lost soon after joining a genome; those that persist are then inherited vertically. This helps to reconcile why gene trees tend to provide valid phylogenetic inferences about the relationships among different bacterial lineages, despite the potential mixing that could result from LGT. Phylogeneticists aiming to reconstruct a phylogeny for a group look at variations in genes distributed in the species, and these are largely vertically transmitted.

Lerat et al. propose that LGT is a common source of genes in γ-Proteobacteria because it has the potential to introduce functionally different genes into the genome with immediate contributions to fitness. Gradual evolution of gene duplicates doesn't provide the same type of immediate reward.

